# Numerical Simulation of Dry Granular Flow Impacting a Rigid Wall Using the Discrete Element Method

**DOI:** 10.1371/journal.pone.0160756

**Published:** 2016-08-11

**Authors:** Fengyuan Wu, Yunyun Fan, Li Liang, Chao Wang

**Affiliations:** Key Laboratory of Ministry of Education on Safe Mining of Deep Metal Mines, Northeastern University, Shenyang, P. R. China; Virginia Commonwealth University, UNITED STATES

## Abstract

This paper presents a clump model based on Discrete Element Method. The clump model was more close to the real particle than a spherical particle. Numerical simulations of several tests of dry granular flow impacting a rigid wall flowing in an inclined chute have been achieved. Five clump models with different sphericity have been used in the simulations. By comparing the simulation results with the experimental results of normal force on the rigid wall, a clump model with better sphericity was selected to complete the following numerical simulation analysis and discussion. The calculation results of normal force showed good agreement with the experimental results, which verify the effectiveness of the clump model. Then, total normal force and bending moment of the rigid wall and motion process of the granular flow were further analyzed. Finally, comparison analysis of the numerical simulations using the clump model with different grain composition was obtained. By observing normal force on the rigid wall and distribution of particle size at the front of the rigid wall at the final state, the effect of grain composition on the force of the rigid wall has been revealed. It mainly showed that, with the increase of the particle size, the peak force at the retaining wall also increase. The result can provide a basis for the research of relevant disaster and the design of protective structures.

## Introduction

In recent years, the outbreaks of rockfall, landslide and debris flow are more frequent which threat to people and infrastructures seriously [1–3]. In these geological disasters, granular flow is one of the typical forms with the characteristics of high flow velocity, long runout distance, huge impact force and bad temporal predictability [4, 5]. In order to reduce the impact of disasters, retaining walls are often used to prevent granular flows [6]. Therefore, it is significant to better understand the mechanism of granular flow impacting a retaining wall.

Experimental research—the most common method—not only can analyze the development mechanism of granular flows, but also can obtain the influencing factors of flow velocity and accumulation shape. For this purpose, Manzella et al. [7] used gravel and small blocks as the tested material to analyze the energy dissipation in the process of sliding down from an inclined board. Inclined chutes have also adopted as the sideway in many experiments [8–11]. Bi et al. [12] revealed complex influences of chutes with different bumpy surface on the velocity and temperature of the granular flows by using two-dimensionally monodisperse disks. Pudasaini et al. [13] performed experiments of dry granular flow impinging an obstructing wall and presented evolution of the height and velocities of both the supercritical and subcritical flows in detail with the granular-PIV measurements on inclined and inclined plus horizontal channel runout. Moreover, granular materials used in experiments were not the same, such as sand [14], ping-pang-ball [15], and glass sphere [16]. Dufresne [17] used coal as avalanche analogue material to study the processes acting well below the surface of a moving rock or debris avalanche during travel over stationary substrate material.

In numerical simulation, granular flows usually can be modeled by either continuum or discrete approaches. In continuous approaches, granular flows have been treated as a Coulomb, or Coulomb-viscoplastic fluid and analyzed by Eulerian forms of continuity and momentum equation [18–23]. In discrete approaches, Discrete Element Method (DEM) as a common numerical method has been widely applied to the simulations of granular flows [24–28]. Numerical verification of laboratory experiments on granular flows down an inclined chute has been presented using DEM [29, 30]. And the force of granular flow impacting rigid obstacles has been further analyzed [31]. Zhou et al. [32] modeled three-dimensional dry granular flows using DEM. The results showed that flow regimes of granular flows can be well identified by combining granular temperature and the Savage number. Utili et al. [33] presented a numerical simulation of dry granular flows generated by the collapse of prismatic columns using DEM in plane strain conditions. Then, energy dissipation of granular flows in dynamic process was analyzed detailedly. Furthermore, combination of DEM and other numerical methods has been carried out, such as DEM-DLM/FD [34], CFD-DEM [35, 36], MFIX-DEM [37]. Manzella et al. [38] used DEM and FEM/DEM to simulate small-scale laboratory experiments which better understand some mechanisms and factors of initial block packing and progressive failure.

The aim of this paper is to investigate the law and mechanism of granular flow impacting a rigid wall using DEM. So far, most of the simulations have been carried out using spherical element. However, real particle shape is complex rather then spherical. Parameter identification and energy dissipation, and the dynamical process are mainly considered in most simulations. Relatively less research on deriving a law and mechanism of granular flow impacting a protective structure has been carried out. So, a better and detailed understanding of the dynamics provides a more comprehensive, accurate and reliable basis for the design of protective structures [3].

## Experimental Set-Up

**Fig 1:**
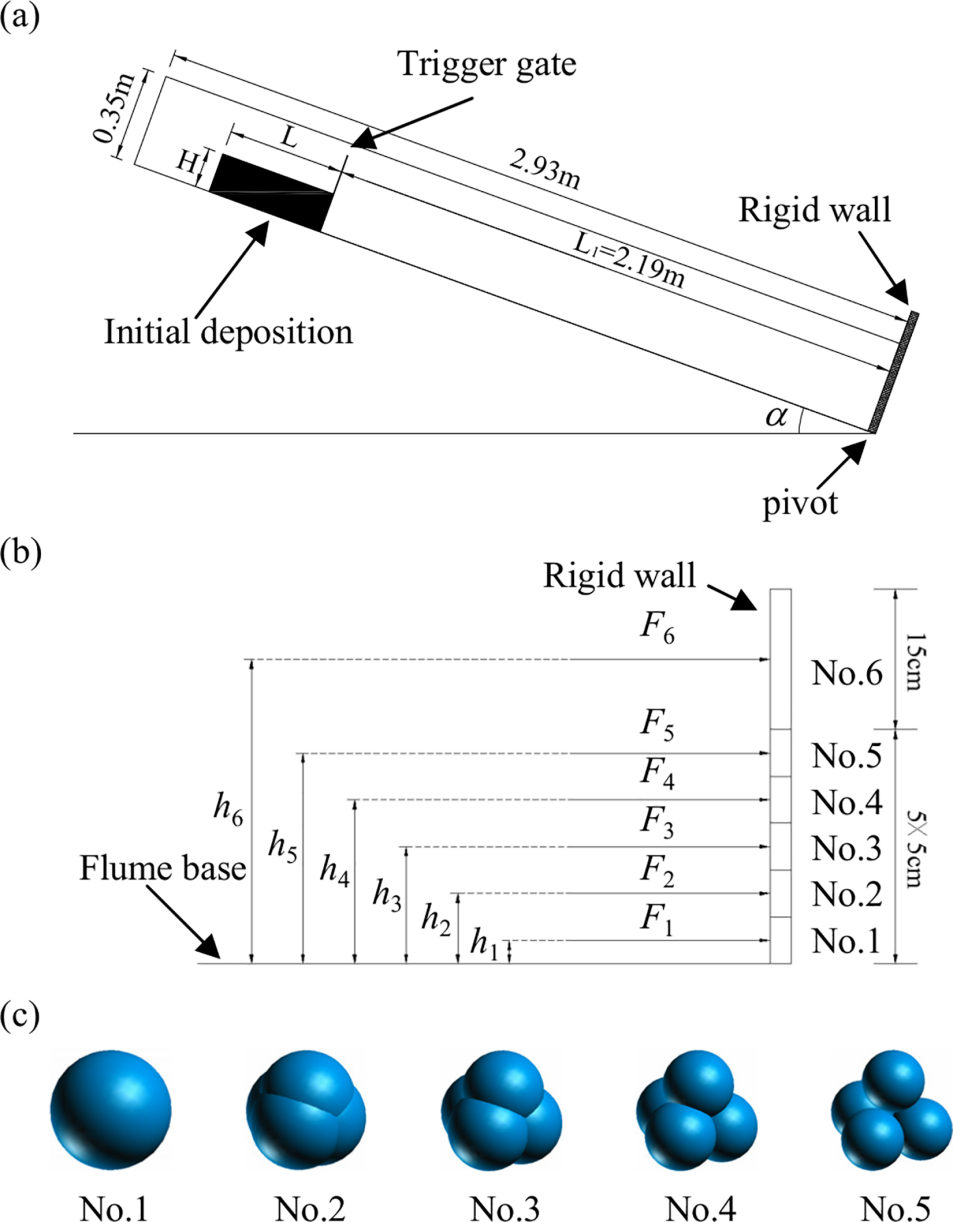
Experimental equipment. (a) Sketch of the experimental flume [39] (b) Sketch of the rigid wall (c) Sketch of the clumps

With 2.93m in length, 0.35m in height, and 0.3m in width, the flume was constructed for the experiment of the granular flows (Fig 1A). The flume was able to rotate around a pivot, and a rigid wall was installed perpendicularly to the flume base at the bottom end so that the normal force could be measured. Tested material was limestone gravel with a specific weight of 13.5kN/m^3^, which had particles ranging from 10 to 20mm in diameter. A trigger gate was used to instigate the flow of the material. As shown in Fig 1A, L was the length, and H was the height of the initial material, and *L*_1_ was the distance between the trigger gate and the rigid frontal wall obstructing the granular flow. Angle *α* was the tilt angle of the flume. The friction angle of the flume base, flume sides (all rigid), and the rigid wall were 25°, 15° and 21°, respectively [39].

The rigid wall was divided into six segments parallel to the channel slope with a width of 300mm. From the first to the sixth segments, the corresponding normal force of the unit width was designated as *F*_*i*_, with *i* being the number of each horizontal segments. *h*_*i*_ was the distance from the centroid of each horizontal segment to the bottom of the rigid wall. As shown in Fig 1B, *h*_1_ to *h*_6_ was 25, 25+50, 25+50+50, 25+50+50+50, 25+50+50+50+50, 25+50+50+50+50+100mm, respectively [39].

## Numerical Modeling

The numerical simulation was carried out using DEM. The spherical element is the basic element in the DEM. Movement and interaction of spherical particles can be modeled directly using DEM. Moreover, it is possible to create clumps with arbitrary shape by attaching two or more particles together. So, it is better to simulate the accumulation and dynamics process of granular flows.

The time step is the calculation cycle of DEM. In every calculation cycle, displacements of each particle can be obtained by repeated application of the law of motion to each particle. Then, contact forces of each particle can be obtained by repeated application of a force-displacement law to each contact which may exist between two balls or between a ball and a wall. The contacts are formed and broken automatically during a calculation cycle.

### Contact-stiffness models

The contact force between two balls or between a ball and a wall [40] is decomposed into a normal component and a shear component. The normal contact force vector is calculated by [40]

$$F_{i}^{n}=K^{n}U^{n}n_{i},$$ (1)

where *K*^*n*^ is the normal stiffness at the contact, *U*^*n*^ denotes overlap which is defined to be the relative contact displacement in the normal direction and *n*_*i*_ is a unit normal vector. The shear elastic force-increment vector is calculated by [40]

$$\Delta F_{i}^{s}=-K^{s}U_{i}^{s},$$ (2)

where *K*^*s*^ is the shear stiffness at the contact and $U_{i}^{s}$ is the shear component of the contact displacement-increment vector.

The contact stiffnesses including normal stiffness and shear stiffness relate the contact forces and relative displacements in the normal and shear directions. The common contact-stiffness model is the linear contact model [40] assuming that the stiffnesses of the two contacting entities act in series. The contact normal secant stiffness is calculated by

$$K^{n}=\frac{k_{n}^{\left[\text{A}\right]}k_{n}^{\left[\text{B}\right]}}{k_{n}^{\left[\text{A}\right]}+k_{n}^{\left[\text{B}\right]}},$$ (3)

and the contact shear tangent stiffness is calculated by

$$K^{s}=\frac{k_{s}^{\left[\text{A}\right]}k_{s}^{\left[\text{B}\right]}}{k_{s}^{\left[\text{A}\right]}+k_{s}^{\left[\text{B}\right]}},$$ (4)

where the superscripts [A] and [B] denote the two entities in contact, the *k*_*n*_ and *k*_*s*_ are the normal stiffness and shear stiffness of the two entities respectively.

Another contact-stiffness model which is a nonlinear contact formulation is defined by shear modulus *G* and Poisson’s ratio *ν* of the two contacting balls. The contact normal secant stiffness is calculated by [40]

$$K^{n}=\left(\frac{2\left\langle G\right\rangle \sqrt{2\tilde{R}}}{3\left(1-\left\langle \nu \right\rangle \right)}\right)\sqrt{U^{n}},$$ (5)

and the contact shear tangent stiffness is calculated by [40]

$$K^{s}=\left(\frac{2{\left({\left\langle G\right\rangle }^{2}3\left(1-\left\langle \nu \right\rangle \right)\tilde{R}\right)}^{1/3}}{2-\left\langle \nu \right\rangle }\right){\left|F_{i}^{n}\right|}^{1/3},$$ (6)

where $\left|F_{i}^{n}\right|$ is the magnitude of the normal contact force. For ball-to-ball contact, the multipliers are given by

$$\tilde{R}=\frac{2R^{\left[\text{A}\right]}R^{\left[\text{B}\right]}}{R^{\left[\text{A}\right]}+R^{\left[\text{B}\right]}},$$ (7)

$$\left\langle G\right\rangle =\frac{1}{2}\left(G^{\left[\text{A}\right]}+G^{\left[\text{B}\right]}\right),$$ (8)

$$\left\langle \nu \right\rangle =\frac{1}{2}\left(\nu^{\left[\text{A}\right]}+\nu^{\left[\text{B}\right]}\right),$$ (9)

and for ball-to-wall contact, the multipliers are given by $\tilde{R}=R^{\left[\text{ball}\right]}$, ⟨*G*⟩ = *G*^[ball]^ and ⟨*ν*⟩ = *ν*^[ball]^.

### Clump model

The basic mass properties of a clump are its total mass *m*, location of the center of mass $x_{i}^{\left[\text{G}\right]}$, and moments and products of inertia *I*_*ii*_ and *I*_*ij*_. For a general clump comprised of *N*_p_ balls, each of which has mass *m*^[p]^, radius *R*^[p]^ and centroid location $x_{i}^{\left[\text{p}\right]}$, the mass properties are defined or calculated by the following equations.

$$m=\sum_{p=1}^{N_{\text{p}}}m^{\left[\text{p}\right]},$$ (10)

$$x_{i}^{\left[\text{G}\right]}=\frac{1}{m}\sum_{p=1}^{N_{\text{p}}}m^{\left[\text{p}\right]}x_{i}^{\left[\text{p}\right]},$$ (11)

$$I_{ii}=\sum_{p=1}^{N_{\text{p}}}\left\{m^{\left[\text{p}\right]}\left(x_{j}^{\left[\text{p}\right]}-x_{j}^{\left[\text{G}\right]}\right)\left(x_{j}^{\left[\text{p}\right]}-x_{j}^{\left[\text{G}\right]}\right)+\frac{2}{5}m^{\left[\text{p}\right]}R^{\left[\text{p}\right]}R^{\left[\text{p}\right]}\right\},$$ (12)

$$I_{ij}=\sum_{p=1}^{N_{\text{p}}}\left\{m^{\left[\text{p}\right]}\left(x_{i}^{\left[\text{p}\right]}-x_{i}^{\left[\text{G}\right]}\right)\left(x_{j}^{\left[\text{p}\right]}-x_{j}^{\left[\text{G}\right]}\right)\right\};\;\left(j\neq i\right),$$ (13)

The motion of a clump can be described in terms of the translational and the rotational motion of the entire clump. The equation for rotational motion can be written in the vector form

$$M_{i}={\dot{H}}_{i},$$ (14)

where *M*_*i*_ is the resultant moment about the center of mass and ${\dot{H}}_{i}$ is the time rate-of-change of the angular momentum of the clump. The resultant moment is calculated by

$$M_{i}=\sum_{p=1}^{N_{\text{p}}}\left({\tilde{M}}_{i}^{\left[\text{p}\right]}+\varepsilon_{ijk}\left(x_{j}^{\left[\text{p}\right]}-x_{j}^{\left[\text{G}\right]}\right)F_{k}^{\left[\text{p}\right]}+\sum_{c=1}^{N_{\text{c}}}\varepsilon_{ijk}\left(x_{j}^{\left[\text{c}\right]}-x_{j}^{\left[\text{p}\right]}\right)F_{k}^{\left[\text{p},\text{c}\right]}\right),$$ (15)

where ${\tilde{M}}_{i}^{\left[\text{p}\right]}$ is the externally-applied moment acting on particle (*p*), $F_{k}^{\left[\text{p}\right]}$ is the resultant force acting on particle (*p*) at its centroid, and $F_{k}^{\left[\text{p},\text{c}\right]}$ is the force acting on particle (*p*) at contact (*c*).

The clump model was used to carry out the numerical simulations, because it was better to model the real limestone gravel. The clump was comprised of four spherical particles with the same diameter. At the same time, each distance between two particles within a clump was the same. Compared with the clump comprised of two or three spherical particles, the clump comprised of four spherical particles held spatial characteristics.

As different distance between the two particles within a clump results in different sphericity of the clump, the clump with better sphericity should be selected. The distance between the two particles was designated as *d*, and the radius of spherical particles was designated as *R*. Five clumps with different sphericity were generated. The relation between *d* and *R* of the five clumps was *d* = 0, *d* = 0.5*R*, *d* = *R*, *d* = 1.5*R* and *d* = 2*R*, respectively. The five clumps which are numbered as 1–5 are shown in Fig 1C, respectively.

In order to calculate the number of clumps needed in the simulations, it is necessary to obtain the quantitative relationship between the volume of a clump and the radius of the spherical particles used in the clump. In clump 1, as *d* = 0, the clump was a sphere with the volume calculated by 4 / 3*πR*^3^. In clump 5, as *d* = 2*R*, the four spherical particles were tangent to each other. So, the volume of clump 5 was equal to sum of the volume of four spherical particles. However, the calculation formulas of the volume of clump 2, clump 3 and clump 4 were more complex. The volumes of clump2, clump3, clump4, which were obtained using curve fitting method as follows: *V*_2_ = 7.4374*R*^3^, *V*_3_ = 11.167*R*^3^, *V*_4_ = 14.7668*R*^3^.

In order to make the volume of the five clumps equal to the volume of the sphere with 20mm in diameter, the radius of spherical particles in each clump should be given as shown in Table 1. At the same time, the specific weight of the clump should be equal to the specific weight of the limestone gravel (taken as 26.5kN/m^3^ for the limestone gravel considered). As clump 1 was a sphere, the specific weight of the spherical particles in clump 1 was 26.5kN/m^3^. As the volume of clump 5 was same to the sum of the volume of four spherical particles, the specific weight of the spherical particles in clump 5 was 26.5kN/m^3^, too. However, as shown in Fig 1C, the volume of clump 2, clump 3 and clump 4 was less than the sum of the volume of the four spherical particles, respectively. According to formula (10), the equation of the specific weight *γ* of spherical particles used in clumps was

$$4\cdot \frac{4}{3}\pi R^{3}\gamma =V_{s}\gamma_{s},$$ (16)

where *V*_*s*_ is the volume of a clump and *γ*_*s*_ is the specific weight of a clump (26.5kN/m^3^). As a result, the specific weight *γ* of spherical particles used in clumps is shown in Table 1.

**Table 1:** Radius and specific weight of spherical particles used in different clumps.

Clump number	1	2	3	4	5
**Radius of spherical particles used in clump(mm)**	10	8.3	7.2	6.6	6.3
**Specific weight of spherical particles used in clump(kN/m**^**3**^**)**	26.5	11.8	17.7	23.4	26.5

Considering that the number of clumps used in the simulations should be similarly equal to the number of material particles in the test, so the number of clumps (*n*_p_) is calculated by [41]

$$n_{\text{p}}=\frac{V_{t}\gamma_{t}}{V_{s}\gamma_{s}},$$ (17)

where *V*_*t*_ is the total volume of the sample, and *γ*_*t*_ is the specific weight of the sample (13.5kN/m^3^).

## Numerical Simulation

In the numerical simulation, three different tests including test L34-H15-α45°, test L44-H20-α40° and test L44-H15-α40° were selected for the model calibration and validation. Tested number indicated the condition of the test. For example, L34-H15-α45° indicated that *L* was 34cm; *H* was 15cm and *α* was 45°.

### Model calibration

In order to select a clump with better sphericity in the five clumps, the numerical simulation for test L34-H15-α45° was carried out using the five clumps. For the convenience of discussion, the force evolution of *F*_1_ was taken as an example.

**Fig 2:**
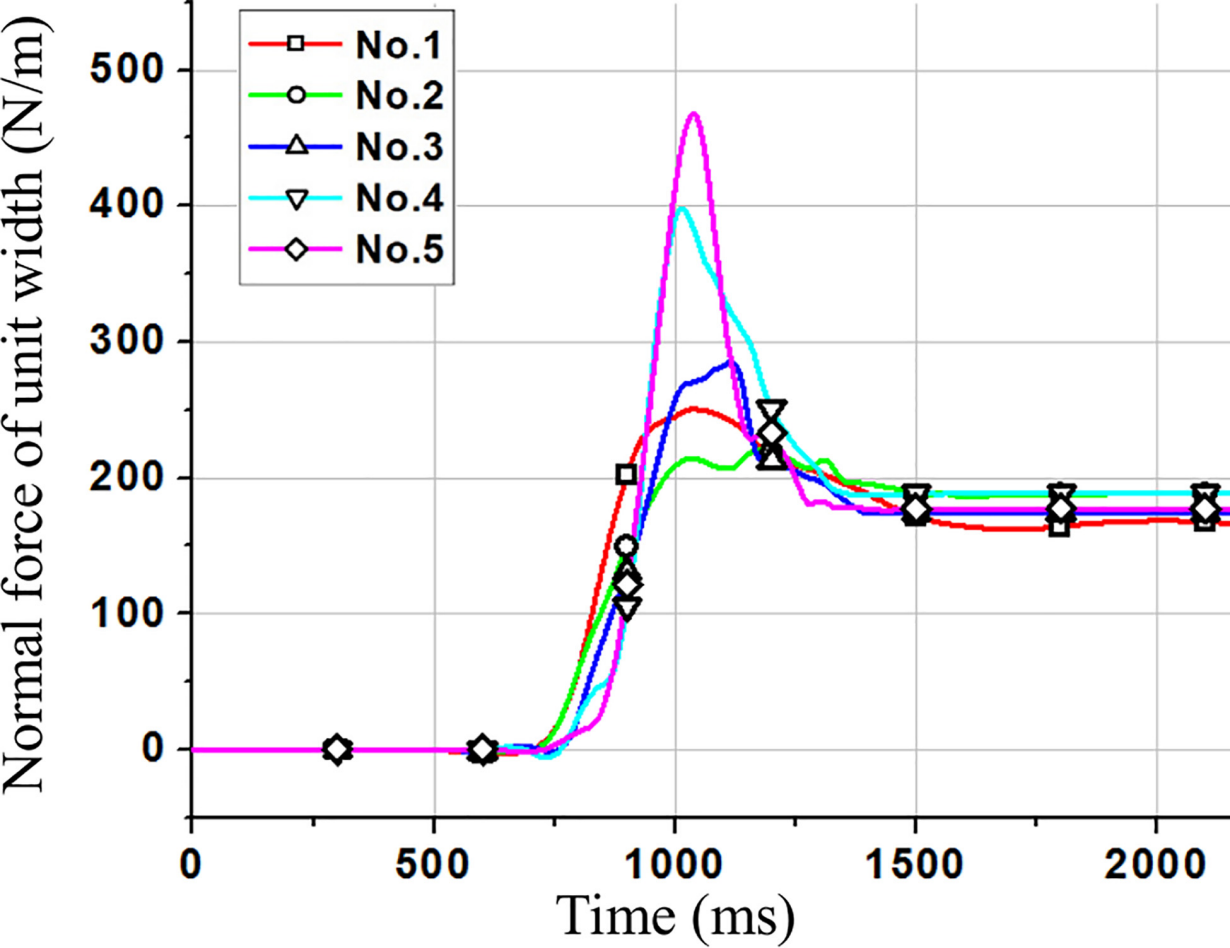
Variation of *F*_1_ with time for the five clumps.

Calculation results of residual and peak of *F*_1_ using the five clumps in Fig 2 are shown in Table 2. The residual value and peak value of *F*_1_ were about 190N/m and 350N/m respectively in tests [40]. The calculation results using clump 4 were more close to the experimental data as shown in Table 2. So the clump 4 was selected to complete other numerical simulations.

**Table 2:** Calculation results of residual and peak of *F*_1_ using the five clumps.

Clump number	1	2	3	4	5
**Residual value of *F***_**1**_**(N/m)**	167	187	174	189	177
**Peak value of *F***_**1**_**(N/m)**	251	221	285	398	468

The calculation results of the time from start to impacting were different from the experimental results. In order to analyze the reason, for test L34-H15-α45°, the tested material was assumed as a rigid body with the same total volume. According to Newton's second law, the time from start to impacting is 1.1s. However, the frictional resistance of the rigid body is sliding frictional resistance which is more than the frictional resistance of the granular flow in test L34-H15-α45° (The frictional resistance of the granular flow may be rolling frictional resistance, and the particles in front are pushed at the rear of the granular flow). Therefore, the time of the granular flow from start to impacting was less than the time of the rigid body, which is consistent with the calculation results. As a result, this is reasonable although simulation results of the time were less than experimental results.

### Model validation

**Fig 3:**
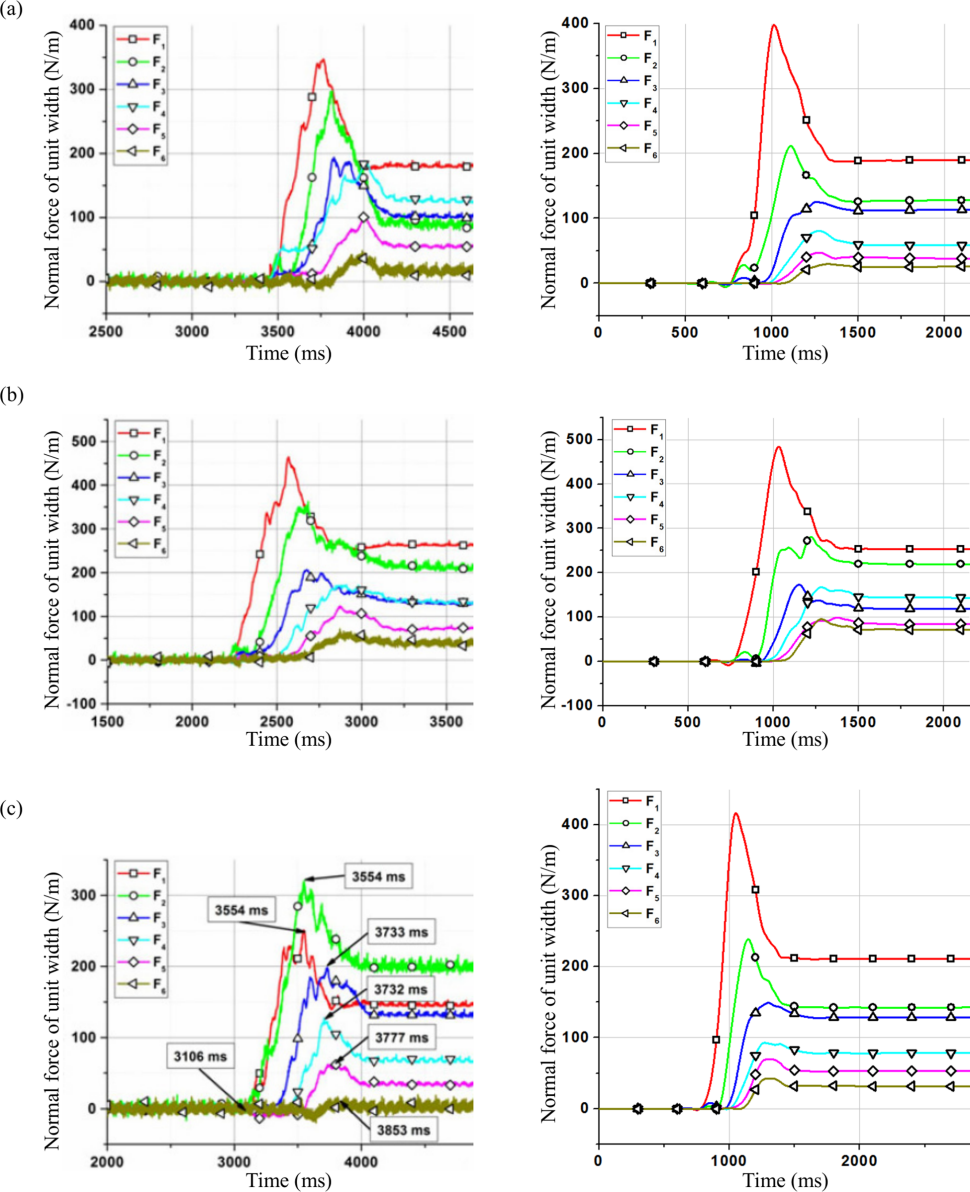
Time history of normal force variation: experiment [40] and model.

According to model calibration, numerical simulations for test L44-H20-α40° and test L44-H15-α40° were carried out using clump 4. The simulation results of normal force are shown in Fig 3. Due to the tendency of DEM results showing large fluctuation, de-noising has been carried out on the DEM results for quantitatively comparable to the experimental data.

In test L34-H15-α45°, the tilt angle of the flume was greater than the tilt angle of the other two flumes. The peak value of *F*_1_ (398N/m) was relatively greater than in the experiment, but with a close residual value in the model (189N/m) compared with the experiment. However, peak and residual value of *F*_4_ in the model were different from experimental values. The residual value of *F*_4_ was greater than the residual values of *F*_2_ and *F*_3_ in the experiment. According to Jiang and Towhata [40], this might be due to a formation of an arch-like protective layer resulting in a non-linear distribution of force with depth. The peak values of *F*_2_, *F*_3_, *F*_5_ and *F*_6_ in the model were 211, 125, 47 and 30N/m respectively along with peak times 1110, 1255, 1272 and 1328ms respectively. Residual values of *F*_2_, *F*_3_, *F*_5_ and *F*_6_ were found to be 128, 113, 38 and 26N/m respectively which are all close to the experimental observations.

For test L44-H20-α40°, the total volume of the sample was greater than the total volume of the samples in the other two experiments. The peak and residual value of *F*_1_ were 484 and 253N/m respectively which are close to the experimental values. The residual value of *F*_2_ (219N/m) was relatively similar to the experiment but with a lower peak value in the model (280N/m) compared with the experiment. For *F*_3_, *F*_4_, *F*_5_ and *F*_6_, the peak values captured by the model were 173, 167, 98 and 95N/m at the times 1151, 1283, 1370 and 1283ms, and the residual values were 118, 144, 85 and 72N/m, which are also close to the experimental values.

In test L44-H15-α40°, the peak values were 416 and 239N/m for *F*_1_ and *F*_2_ respectively which were not similar to the experiment values. Such discrepancy of the force evolution was due to a non-linear distribution of force with depth as discussed in test L34-H15-α45°. Concerning the rest of the wall, the *F*_3_, *F*_4_, *F*_5_ and *F*_6_ achieved peak values 149, 93, 70 and 43N/m respectively at the times 1301, 1273, 1323 and 1327ms respectively, and the residual values were 128, 78, 53 and 32N/m which are in relatively good agreement with the experimental results. However, the impact times do not correspond.

**Fig 4:**
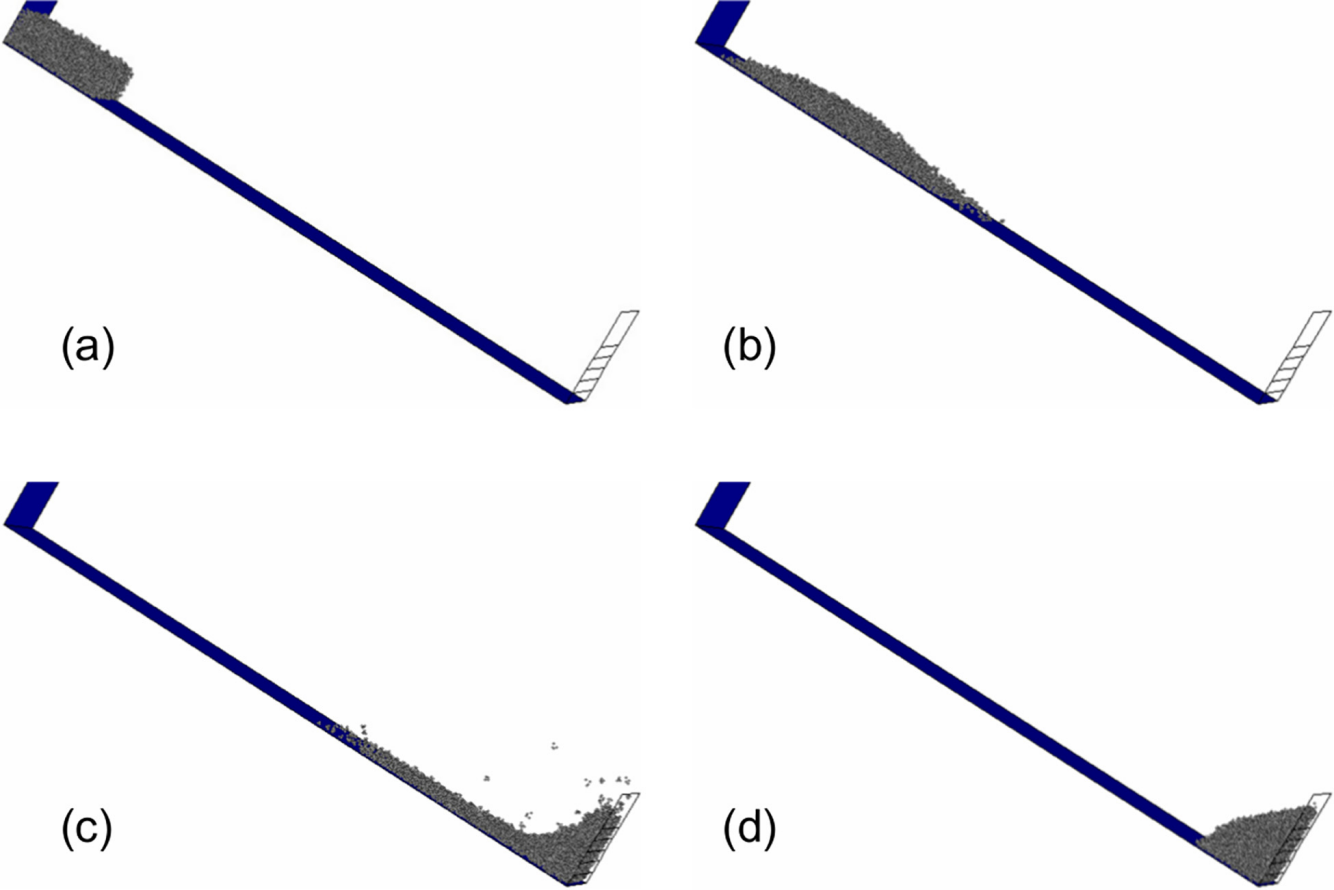
Snapshots of the evolution of simulated flow through time for test L44-H15-α40°. (a) at time = 0ms (b) at time = 500ms (c) at time = 1200ms (d) at time = 2300ms

According to the comparison results, although calculation results have some differences from experimental data in local details, the simulations of motion process of the granular flow and force evolution of the rigid wall have been carried out in general. In order to investigate the features in the process of flowing, for test L44-H15-α40°, snapshots of the evolution of simulated flow at several typical moments are shown in Fig 4. Fig 4A showed the accumulation form of the granular flow at the initial moment. Then the granular flow was released instantaneously when the trigger gate was taken away. Fig 4B was the calculation result which showed the accelerated development status of the granular flow. The accumulation form of the granular flow was flat and the deformation effect lowered the centre of gravity of the granular flow. In Fig 4C, the granular flow impacted the rigid wall. The particles in the front of the granular flow deposited behind the bottom of the rigid wall after impacting. The particles at back of the granular flow performed upward movement as the particles in front providing a boundary-layer [13]. The shape of the granular flow appeared concave upward. Fig 4D showed the final accumulation form of the granular flow. In the later stage of the motion, due to the embedding of the particles at back of the granular flow and downward movement of the upper unstable particles, the accumulation surface formed a convex shape.

### Total normal force and bending moment

The total force and bending moment acting on the wall were calculated by

$$F=\sum_{i=1}^{6}F_{i},$$ (18)

$$M=\sum_{i=1}^{6}F_{i}h_{i},$$ (19)

**Fig 5:**
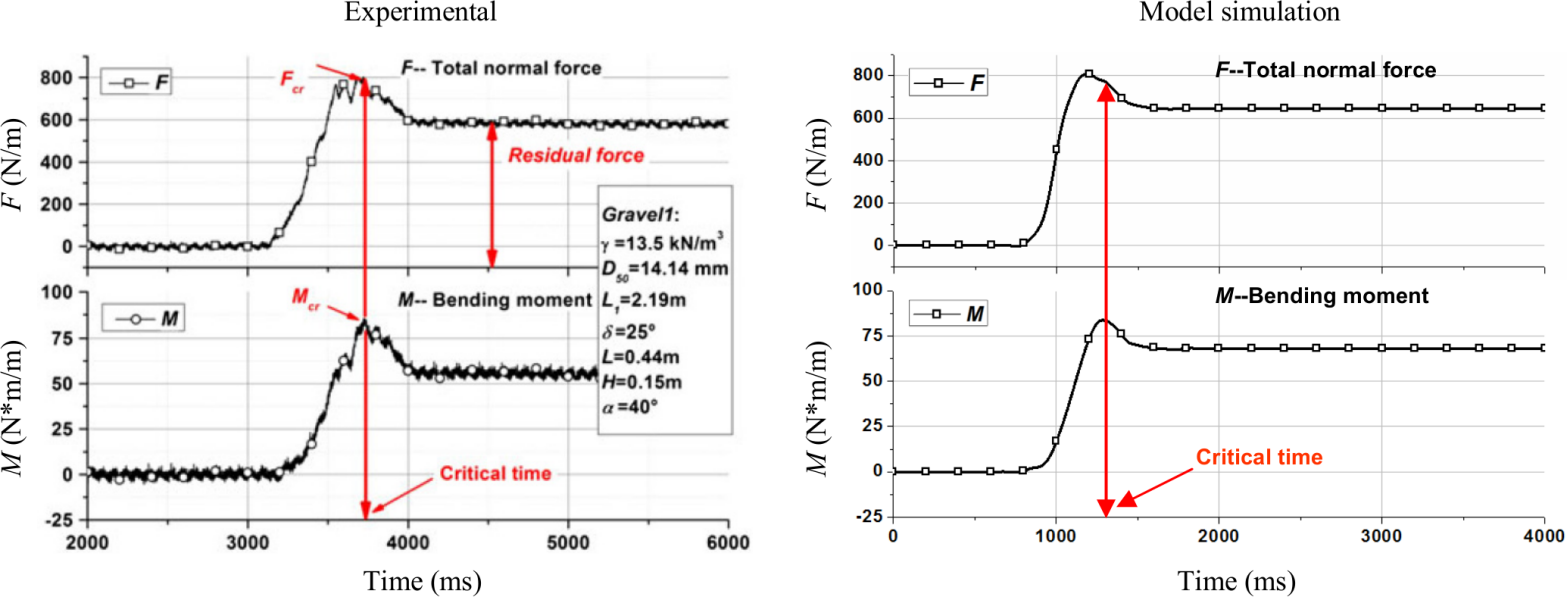
Time history of total normal force and bending moment for test L44-H15-α40°: experiment [40] and numerical model.

Time history of total normal force and bending moment of experiment and numerical model for test L44-H15-α40° are shown in Fig 5. The peak and residual value of F are 807 and 645N/m, respectively, and the peak and residual value of M are 84 and 68N*m/m, respectively. All these values fairly agree with the experimental results. However, although the critical times are in good agreements, the absolute impact times do not match.

In general, not only the normal force at each part of the wall, but also the total normal force and bending moment in simulations agree well with experimental results. This verifies the effectiveness of the present model.

### Granular flows with grain composition

In reality, the granular flow is not composed of particles with the same diameter but composed of particles with different grain sizes, called composition. In order to analyze the influence on the force at the rigid wall by grain composition, numerical simulations for test L44-H15-α40° were carried out using three clumped groups with different simple grain composition. For simplicity, each of clumped groups is assumed to have the same total weight which is equal to the weight of the sample composed of clumps with the same diameter. The mass fractions of clumps with different diameter in three groups are shown in Table 3.

**Table 3:** Mass fraction of clumps with different diameter in three groups.

**Clumped group 1**						
**Diameter(mm)**	10		20		40	
**Mass fraction**	25%		50%		25%	
**Clumped group 2**						
**Diameter(mm)**	15		20		30	
**Mass fraction**	25%		50%		25%	
**Clumped group 3**						
**Diameter(mm)**	10	15		20	30	40
**Mass fraction**	5%	20%		50%	20%	5%

**Fig 6:**
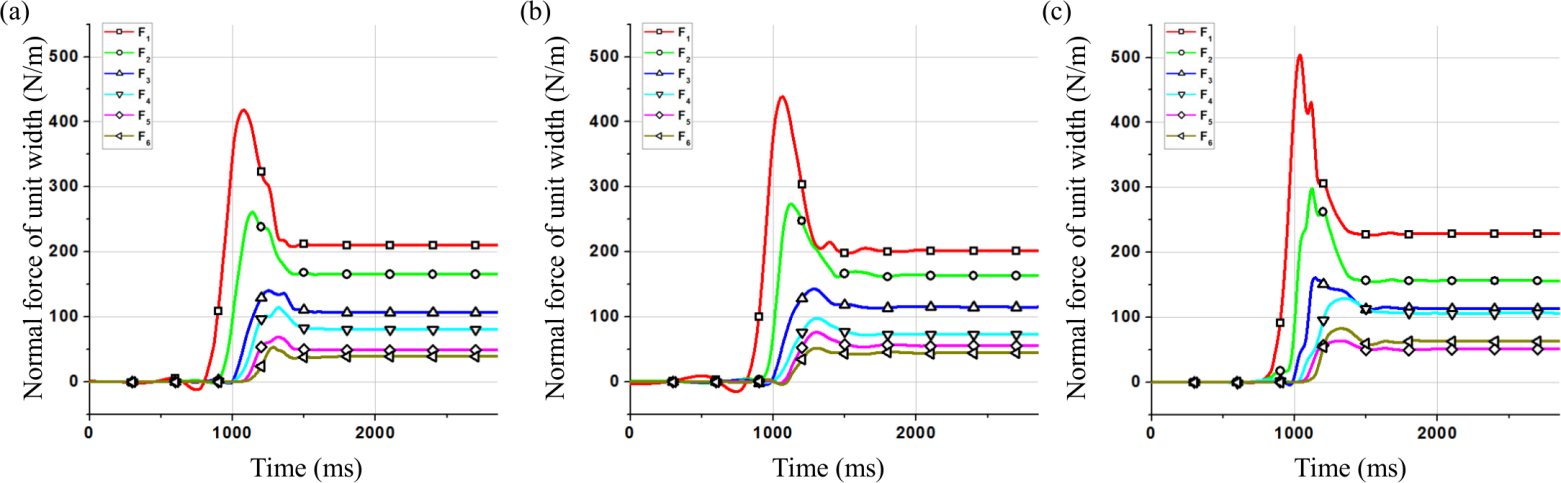
Time history of normal force of different groups for test L44-H15-α40°. (a) Clumped group 1 (b) Clumped group 2 (c) Clumped group 3

**Fig 7:**
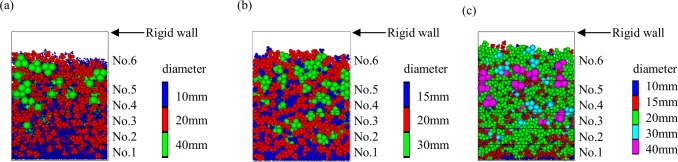
Distribution of the particles in final deposition behind the wall. (a) Clumped group 1 (b) Clumped group 2 (c) Clumped group 3

As shown in Fig 6, compared with the calculation results of peak forces using the sample composed of clumps with the same diameter in model validation, the peak values of *F*_1_ and *F*_2_ change obviously. As shown in Table 4, when the particle diameter is not the same, the peak values of *F*_1_ and *F*_2_ are higher. The residual value of *F*_6_ changes which has the same trend with the peak values of *F*_1_ and *F*_2_. In order to analyze this effect, the distribution of the clumps in the final deposition behind the wall is shown in Fig 7.

**Table 4:** Calculation results of part of peak and residual values of different groups.

	clumps with the same diameter	Clumped group 1	Clumped group 2	Clumped group 3
**Peak value (N/m) of *F***_**1**_	416	418	438	504
**Peak value (N/m) of *F***_**2**_	239	261	274	298
**Residual value (N/m) of *F***_**6**_	31	39	44	63

As shown in Fig 7, the clumps with larger diameter are mostly distributed at the upper part of the granular flow, such as clumps with 40mm diameter in Fig 7A, 30mm diameter in Fig 7B and clumps with 40mm diameter in Fig 7C. However, compared with the clumps with larger diameter, the clumps with smaller diameter deposit at the bottom of the granular flow. The appearance of particles reverse separation might lead to the change of *F*_6_. When the particles impact the rigid wall, the impacting of larger clumps causes a greater normal force. This phenomenon is probably caused by the dispersion impact forces of larger clumps, which are separated reversely to the upper granular flow, while the smaller clumps are mainly concentrated at the bottom of the deposition layer of the granular flow.

**Fig 8:**
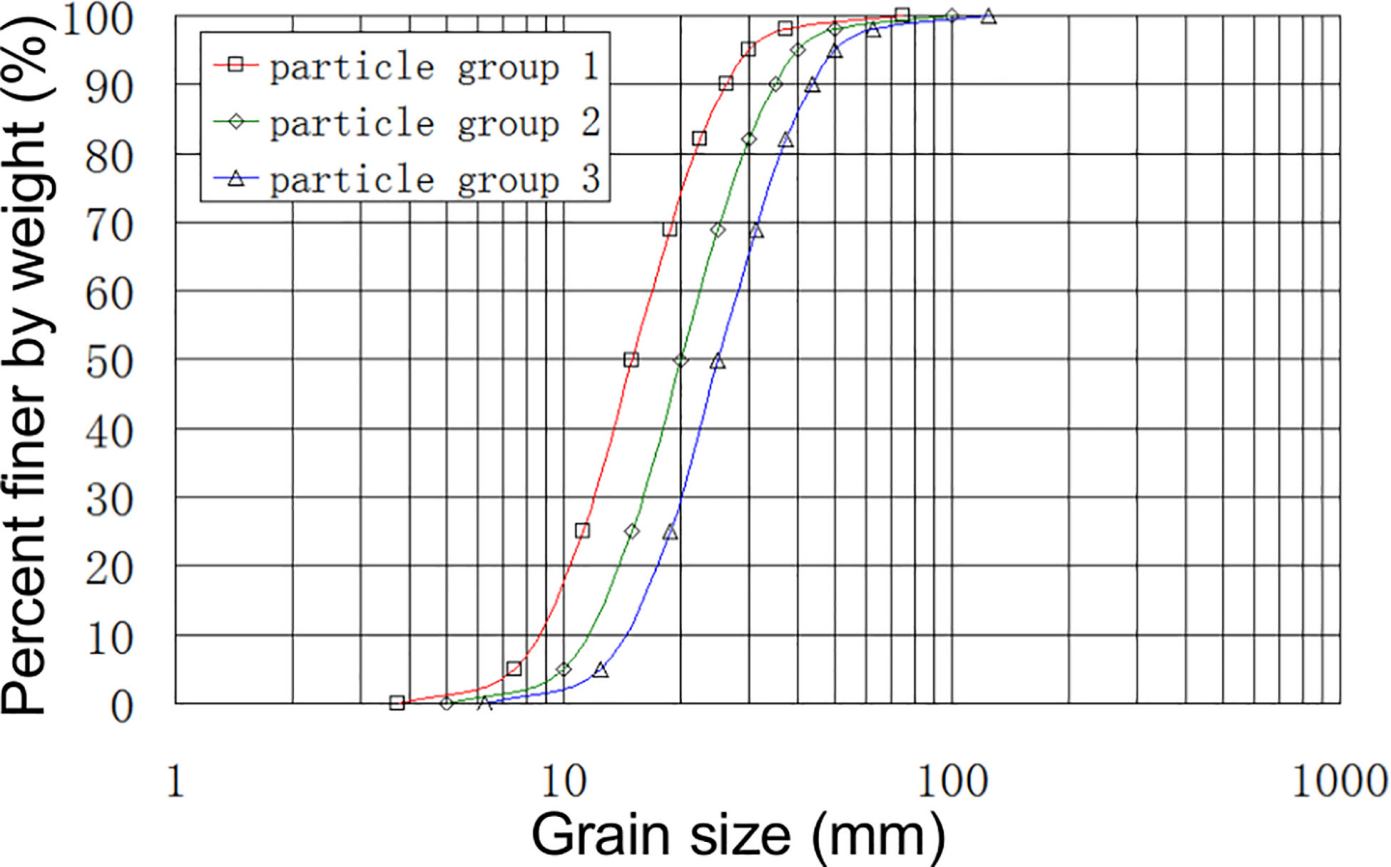
Particle grading curve.

Therefore, the grain composition has an effect on the distribution of the force of a rigid wall. For further analysis, numerical simulations for test L44-H15-α40° were carried out using three particle groups with different complex grain composition. The median diameter of the three particle groups were 15, 20 and 25mm. The particle grading curve is shown in Fig 8. Taking into account the influence of computational efficiency, spherical element was used in the simulations. At the same time, numerical simulations were carried out using the particles with the same diameter of 15, 20 and 25mm, respectively.

**Fig 9:**
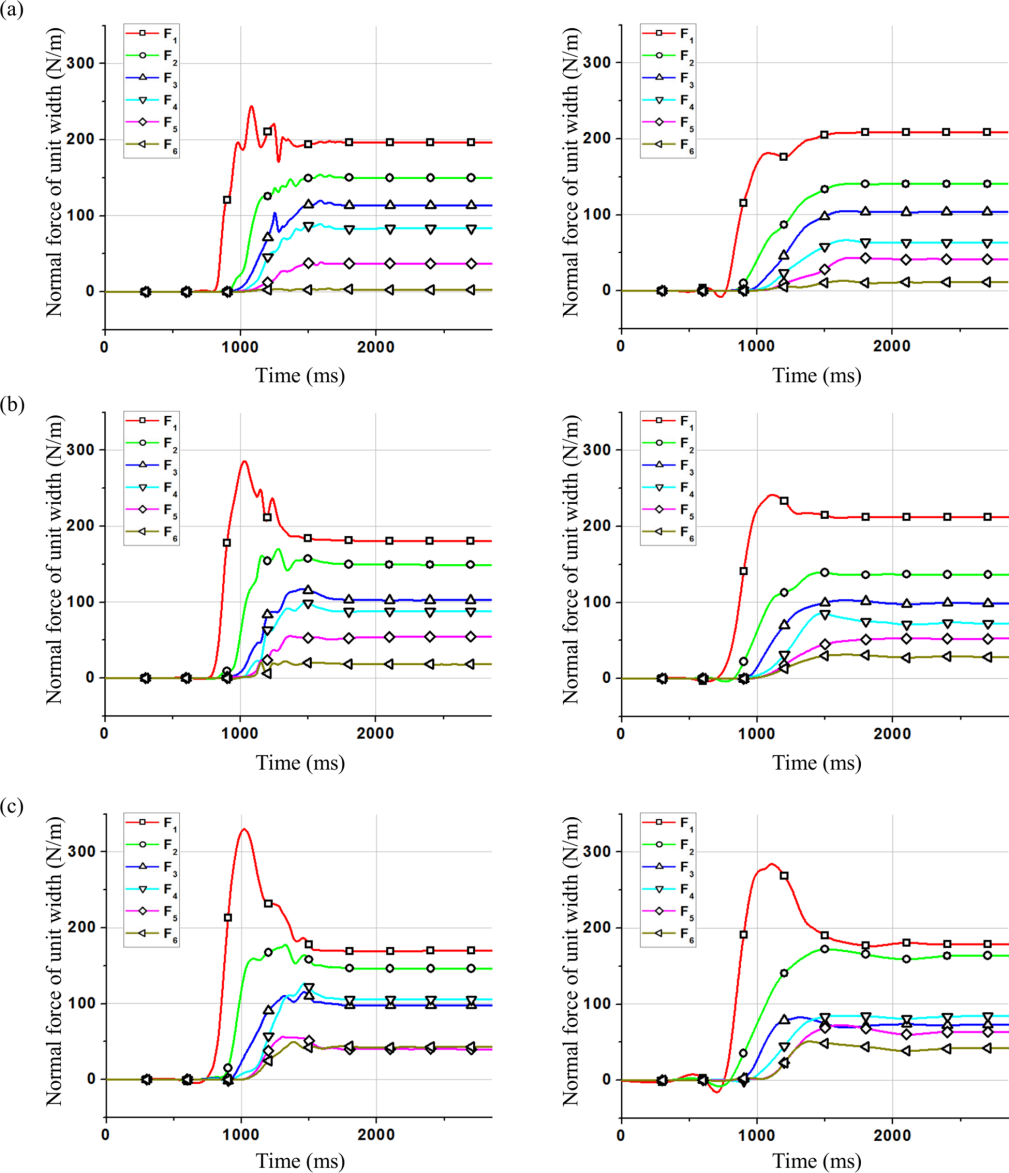
**Calculation results of normal force on the rigid wall using the three particle groups with different complex grain compositions (*left*) and the particles with the same diameter which are the median diameter of the three particle groups (*right*).** (a) particle group 1 (b) particle group 2 (c) particle group 3

As shown in Fig 9, calculation results of peak force on the rigid wall become greater when using the particles with complex grain composition than that when using the particles with the same diameter which are the median diameter of the three particle groups. The peak value of the normal force increases when the particle diameter increases, with the most significant increase for *F*_1_.

This analysis shows that, the influence of grain composition should be considered on the structural design of the retaining wall, especially at the bottom of the retaining wall. This effectively reduces the potential damage and loss caused by granular flows.

## Conclusions

In this paper, we have carried out numerical simulations of the impact of dry granular flow against a rigid wall using DEM. In the simulations, we have developed a clump model which performs better than spherical ones. Satisfactory agreement has been observed in terms of the normal force of each part of the wall, the total normal force, and bending moment between the model and experiment, which indicates the correctness and effectiveness of the model.

In the comparison among the calculation results using the groups with different clump diameter and the calculation results using the clumps with the same diameter, it is shown that the particle diameter distribution is wider, and then the peak force of the wall is greater. This phenomenon is mainly the result of reverse separation in the motion process of the granular flow.

According to the actual grain composition of granular flows, we have carried out the simulation analysis of granular flows with complex grain composition and the particles with the same diameter which is equal to the median diameter. It indicates that the increase of peak force of the wall is due to the increase of particle diameter.

This paper presents the law and mechanism of dry granular flow impacting a rigid wall effectively which promotes the understanding of the granular flow against a retaining structure and provides a reference and basis for the engineering structure design to prevent and mitigate disasters.

## Supporting Information

S1 File Data of Fig 2. (XLS)

S2 File Data of Fig 3. (XLS)

S3 File Data of Fig 5. (XLS)

S4 File Data of Fig 6. (XLS)

S5 File Data of Fig 8. (XLS)

S6 File Data of Fig 9. (XLS)
